# Ethyl Rosmarinate Protects High Glucose-Induced Injury in Human Endothelial Cells

**DOI:** 10.3390/molecules23123372

**Published:** 2018-12-19

**Authors:** Yan-Hui Shen, Li-Ying Wang, Bao-Bao Zhang, Qi-Ming Hu, Pu Wang, Bai-Qiu He, Guan-Hu Bao, Jing-Yu Liang, Fei-Hua Wu

**Affiliations:** 1 Department of Pharmacology of Chinese Materia Medica, School of Traditional Chinese Pharmacy, China Pharmaceutical University, Nanjing 211198, China; m18851109778@163.com (Y.-H.S.); wangliying_1988@163.com (L.-Y.W.); song212810@163.com (B.-B.Z.); wpcpu438@163.com (P.W.); hbqnjfu@163.com (B.-Q.H.); 2 Jiangsu Key Laboratory of TCM Evaluation and Translational Research, School of Traditional Chinese Pharmacy, China Pharmaceutical University, Nanjing 211198, China; 3 Department of Natural Medicinal Chemistry, School of Traditional Chinese Pharmacy, China Pharmaceutical University, Nanjing 211198, China; jyliang08@126.com; 4 Natural Products Laboratory, State Key Laboratory of Tea Plant Biology and Utilization, Anhui Agricultural University, Hefei 230036, China; huhu1995@126.com (Q.-M.H.) baoguanhu@ahau.edu.cn (G.-H.B.)

**Keywords:** ethyl rosmarinate, vascular endothelial cell, apoptosis, high glucose, rosmarinic acid

## Abstract

Ethyl rosmarinate (RAE) is one of the active constituents from *Clinopodium chinense* (Benth.) O. Kuntze, which is used for diabetic treatment in Chinese folk medicine. In this study, we investigated the protective effect of RAE on high glucose-induced injury in endothelial cells and explored its underlying mechanisms. Our results showed that both RAE and rosmarinic acid (RA) increased cell viability, decreased the production of reactive oxygen species (ROS), and attenuated high glucose-induced endothelial cells apoptosis in a dose-dependent manner, as evidenced by Hochest staining, Annexin V–FITC/PI double staining, and caspase-3 activity. RAE and RA both elevated Bcl-2 expression and reduced Bax expression, according to Western blot. We also found that LY294002 (phosphatidylinositol 3-kinase, or PI3K inhibitor) weakened the protective effect of RAE. In addition, PDTC (nuclear factor-κB, or NF-κB inhibitor) and SP600125 (c-Jun N-terminal kinase, or JNK inhibitor) could inhibit the apoptosis in endothelial cells caused by high glucose. Further, we demonstrated that RAE activated Akt, and the molecular docking analysis predicted that RAE showed more affinity with Akt than RA. Moreover, we found that RAE inhibited the activation of NF-κB and JNK. These results suggested that RAE protected endothelial cells from high glucose-induced apoptosis by alleviating reactive oxygen species (ROS) generation, and regulating the PI3K/Akt/Bcl-2 pathway, the NF-κB pathway, and the JNK pathway. In general, RAE showed greater potency than RA equivalent.

## 1. Introduction

Diabetes is a group of metabolic disorders in which there are high blood sugar levels over a prolonged period [1], which can cause many complications including cardiovascular disease, stroke, chronic kidney disease, foot ulcers, damage to the eyes, etc. [2]. More than half of diabetic patients mainly die because of cardiovascular diseases [3]. Vascular endothelial dysfunction is recognized as initial step of diabetic vascular complications, which might be caused by hyperglycemia-induced vascular endothelial cells apoptosis [4].

A number of signaling pathways have been demonstrated to participate in apoptosis induced by high glucose, including the phosphatidylinositol 3-kinase (PI3K)/Akt pathway, nuclear factor-κB (NF-κB) pathway, and c-Jun N-terminal kinase (JNK) pathway [5,6,7]. Several studies have shown that the PI3K/Akt pathway acts as a negative regulator of apoptosis, and its disruption leads to apoptosis [8,9]. PI3K/Akt activation protects cells from apoptosis by promoting the expression of Bcl-2 and then suppressing the expression of Bax [10]. Additionally, NF-κB is closely related to diabetes complications, which is activated by a variety of physiological or pathological stimuli, such as high glucose [11]. Moreover, the JNK pathway is also involved in the regulation of endothelial cells apoptosis in response to high glucose [9]. Several studies have demonstrated that JNK/NF-κB signaling mediates reactive oxygen species (ROS)-induced apoptosis under high-glucose conditions [12]. Importantly, ROS is reported to be a key component involved with apoptosis. Previous studies have shown that hyperglycemia enhances free radical production and induces oxidative damage, which in its turn activates the cell death pathways associated with apoptosis and necrosis [12,13]. Thus, exploring the relationship between drugs and these pathways involved with high glucose-induced apoptosis and finding potential drug targets may contribute to develop new agents of anti-diabetic vascular complications.

*Clinopodium chinense* (Benth.) O. Kuntze (CC) belongs to the family Labiatae. Its aerial part, which is called “duan xue liu”, is used as a traditional Chinese medicinal material in the Chinese pharmacopoeia [14]. It effectively cures different hemorrhages in clinic, and is used for the treatment of diabetes in Chinese folk. CC was proved to be cytoprotective on vascular endothelial cells induced by high glucose in our previous study [15]. Ethyl rosmarinate (RAE) is an active component in CC with α-glucosidase inhibition and cytoprotection [16]. It has been reported that RAE exhibited the most potent inhibitory effect on NO production in lipopolysaccharide-induced murine alveolar macrophage cells [17], and RAE induced relaxation in aortic rings via an endothelium-independent pathway [18]. In addition, RAE shows great efficiency in inhibiting T cell proliferation, suppressing IL-2 production, and inhibiting ROS production [19]. RAE is an ester derivative of rosmarinic acid (RA), which has been proved to have vascular protective activity [20], as well as antioxidant [21], anti-inflammatory [22], and anti-diabetes effects in the last decade [23]. In our present study, we examined the protective effects of RAE and RA on ROS generation and apoptosis in vascular endothelial cells exposed to high glucose. We also detected the expression of apoptotic pathway-involved proteins including Akt, NF-κB, and JNK to explore the underlying molecular mechanisms of RAE.

## 2. Results

### 2.1. Effect of RAE on Cell Viability Induced by High Glucose

**Figure 1:**
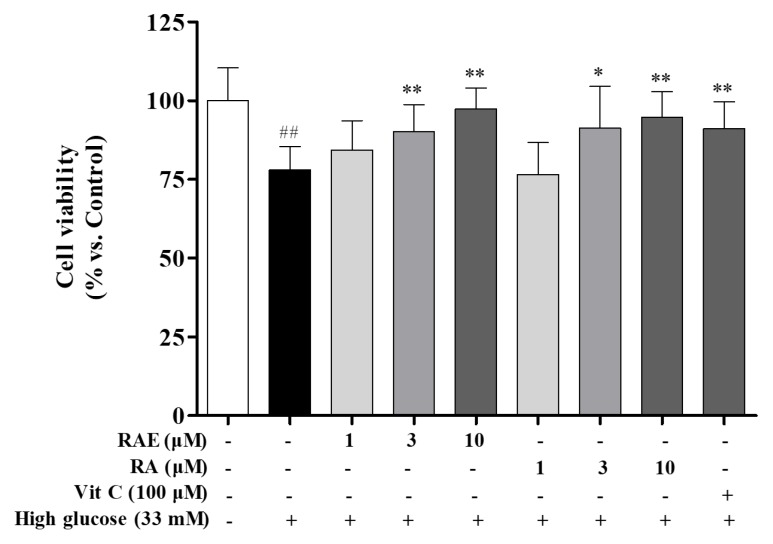
Effect of ethyl rosmarinate (RAE) and rosmarinic acid (RA) on cell viability in high glucose-induced human endothelial cells. EA.hy926 cells were treated with RAE (1, 3, and 10 μM), RA (1, 3, and 10 μM) or positive control Vit-C (100 μM), respectively, in the medium containing 33 mM of glucose for 72 h. The results were expressed as mean ± SD (n = 3). ^##^ *P* < 0.01, vs. control; ^*^ *P* < 0.05, ^**^ *P* < 0.01, vs. high glucose.

We evaluated the effects of RAE on endothelial cells viability using 3-(4,5-dimethylthiazol-2yl-)-2,5- diphenyltetrazoliumbromide (MTT) assay. As shown in Figure 1, compared with the control group, the model group treated with 33 mM of glucose resulted in a significant decrease in cell viability after incubating for 72 h. Treatment with RAE (3 and 10 μM) and RA (3 and 10 μM) markedly prevented endothelial cells from high glucose-induced damage. Treatment of RAE (10 μM) achieved a maximum protective effect (97.3% versus 78.0% viability of the 33-mM glucose group). The positive control group Vitamin C (Vit-C 100 μM) showed a similar protective effect, and the cell viability was 91.0%.

### 2.2. Effect of RAE on ROS Generation in Human Endothelial Cells Induced by High Glucose

**Figure 2:**
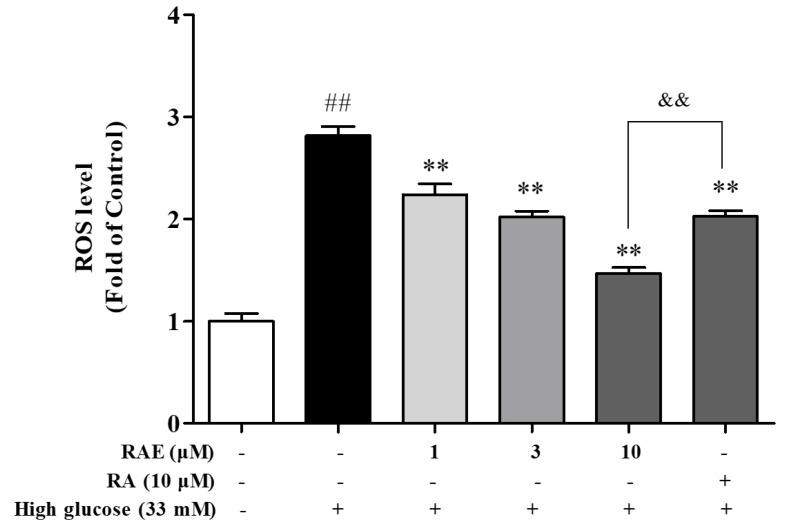
Effect of RAE on ROS generation in high glucose-induced human endothelial cells. EA.hy926 cells were co-treated with 33 mM of glucose and RAE or RA at different concentrations for 48 h. Intracellular ROS production was assessed by fluorescence of 2′,7′-dichlorofluorescin diacetate (DCFH-DA), as described in methods. Results were expressed as mean ± SD (n = 3). ^##^ *P* < 0.01, vs. control; ^**^ *P* < 0.01, ^*^ *P* < 0.05, vs. high glucose; ^&&^ *P* < 0.01, vs. RAE group.

The mitochondrial oxidative stress response to hyperglycemia is the key initiator for endothelial cell apoptosis [13]. Therefore, we evaluated the effect of RAE on ROS production in EA.hy926 endothelial cells exposed to high glucose. As illustrated in Figure 2, the intracellular ROS level in endothelial cells incubated with 33 mM of glucose was 2.8-fold greater than that observed in untreated cells. Treatment with RAE (1, 3, and 10 μM) and RA (10 μM) inhibited the overproduction of ROS induced by high glucose, and the inhibition rates were 31.8%, 43.9%, 74.3%, and 43.5% respectively. RAE decreased the ROS level in a concentration-dependent way. The treatment of RA (10 μM) was less effective than the treatment of RAE (10 μM).

### 2.3. Effects of RAE on Cell Apoptosis Induced by High Glucose

**Figure 3:**
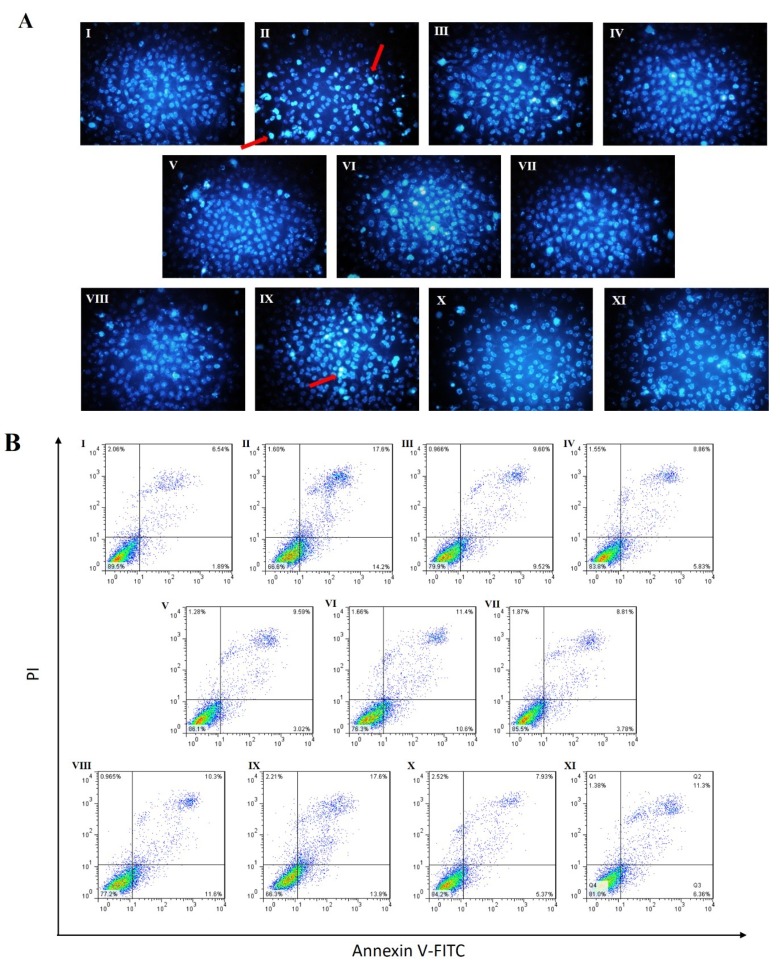

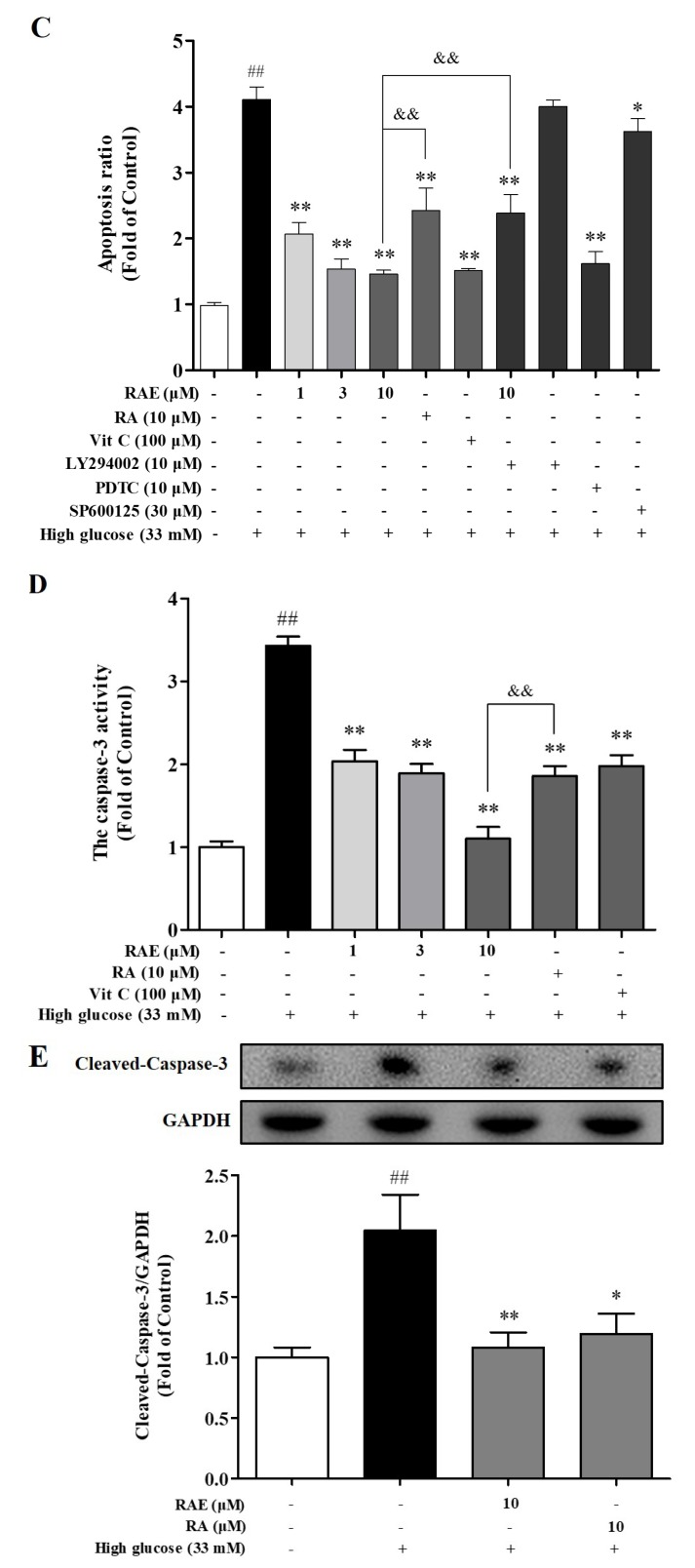
Effect of RAE on apoptosis in human endothelial cells treated with high glucose. EA.hy926 were treated with RAE or RA at indicated concentrations in the medium containing 33 mM of glucose for 48 h. (I) Control; (II) High-glucose model group; (III) RAE 1 μM; (IV) RAE 3 μM; (V) RAE 10 μM; (VI) RA 10 μM; (VII) Vit-C 100 μM; (VIII) LY294002 10 μM + RAE 10 μM; (IX) LY294002 10 μM; (X) PDTC 10 μM; and (XI) SP600125 30 μM. (**A**) Cells were harvested and stained with Hoechst 33258. The staining showed strong blue fluorescence in apoptotic cells, while the normal cells only showed weak fluorescence, and the dead cells were not stained. One representative image of three individual experiments is shown. (**B**) Apoptotic cells were assessed by flow cytometry following Annexin V-FITC/PI staining, according to the manufacturer’s protocol. (**C**) Quantitative analysis of the data from (B) to give the percent of apoptosis cells by flow cytometry. (**D**) Caspase-3 activity was measured using a caspase-3 colorimetric assay kit following the manufacturer’s instructions. (**E**) The expression of cleaved caspase-3 was analyzed with Western blotting. Human umbilical vein endothelial cell lines (HUVEC) cells were treated with or without RAE or RA in the medium containing 33 mM of glucose for 48 h. Cleaved caspase-3 was determined relative to glyceraldehyde-3-phosphate dehydrogenase (GAPDH). One representative image of three individual experiments is shown. The results were presented as mean ± SD (n = 3). ^##^ *P* < 0.01, vs. control; ^*^ *P* < 0.05, ^**^ *P* < 0.01, vs. high glucose; ^&&^ *P* < 0.01, vs. RAE (10 μM) group.

To detect the effect of RAE on high glucose-induced apoptosis in human endothelial cells, fluorescence microscopy with Hochest staining and flow cytometric methods with Annexin V-FITC/PI staining were used. As shown in Figure 3A, after treatment with 33 mM of glucose for 48 h, densely stained with bright blue were significantly exhibited in chromatin in the model group. Meanwhile, densely stained cells were reduced when treated with RAE (1, 3, and 10 μM), RA (10 μM), and Vit-C. A significant increase of apoptotic cells showed in flow cytometry under high glucose stimulation (Figure 3B,C). Treatments of RAE (1, 3, and 10 μM), RA (10 μM), and Vit-C (100 μM) reduced apoptosis, and the inhibition rates were 65.7%, 82.8%, 85.1%, 54.2%, and 83.5%, respectively (Figure 3C). Based on the results, we also found that the protect effect of RAE on endothelial cells apoptosis induced by high glucose was better than RA at the concentration of 10 μM.

When treated with LY294002 (PI3K inhibitor), the apoptosis rate was as high as the model group, as shown in Figure 3C. Moreover, when endothelial cells were treated with LY294002 and RAE (10 μM), the apoptosis rate of cells was significantly increased by 1.6-fold compared with treatment with RAE (10 μM) alone (Figure 3C). These indicated that PI3K/Akt signaling was involved in the apoptosis pathway caused by high glucose, and RAE could reverse the effect of LY294002 on endothelial cells. In addition, when cells were incubated with PDTC (NF-κB inhibitor) or SP600125 (JNK inhibitor), the apoptosis rate was reduced.

Caspase-3 is essential in regulating cell apoptosis as the executive protein. The incubation of high glucose caused a 3.4-fold excessive increase in caspase-3 activity (Figure 3D) in endothelial cells. Compared with the model group, treatment of RAE (1, 3, and 10 μM) and RA (10 μM) led to a significant decrease in caspase-3 activity, and the inhibition rates were 57.5%, 63.4%, 96.2%, and 65.0%, respectively. The results indicated that RAE exerted an anti-apoptotic role in EA.hy926 cells induced by high glucose.

To further validate the anti-apoptotic effect of RAE, Western blotting was performed, as shown in Figure 3E. The results showed that high glucose promoted the cleavage of caspase-3. When treated with RAE or RA, the expression of cleaved-caspase-3 decreased significantly. These suggested that RAE and RA have a protective effect on endothelial cells apoptosis caused by high glucose.

### 2.4. Effect of RAE on PI3K/Akt Signal Pathway

**Figure 4:**
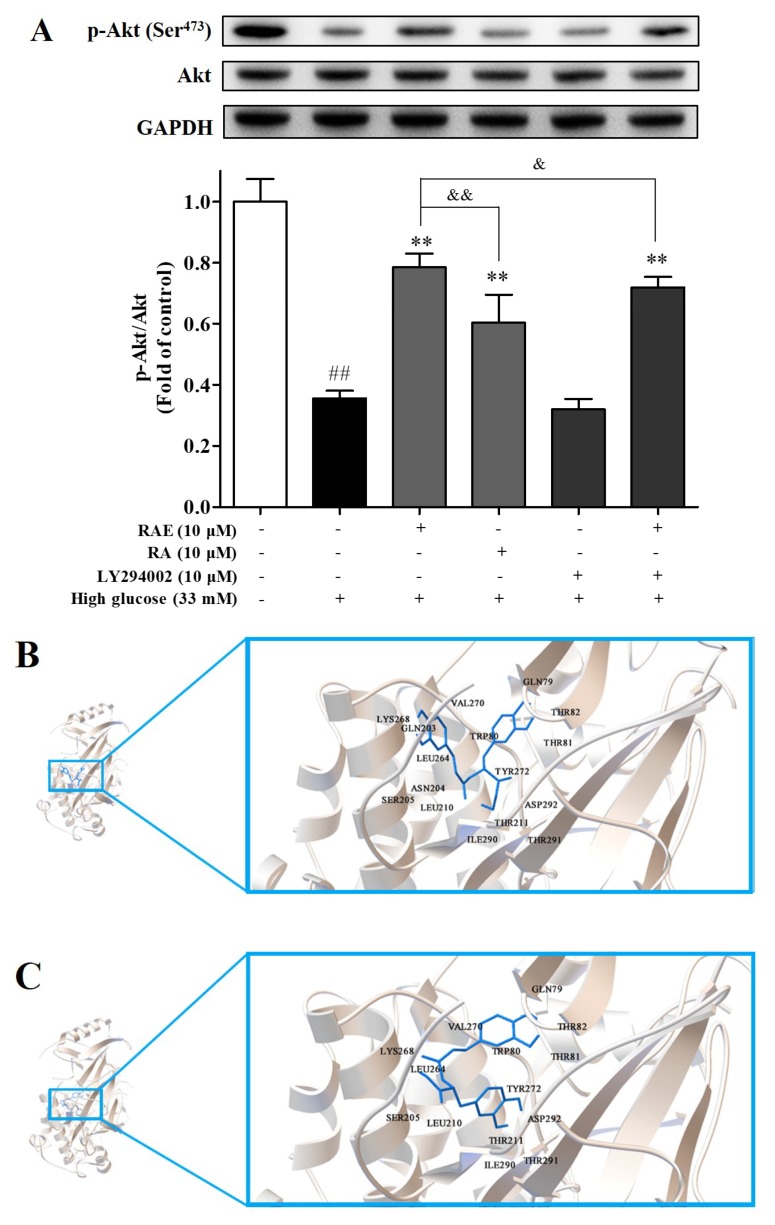

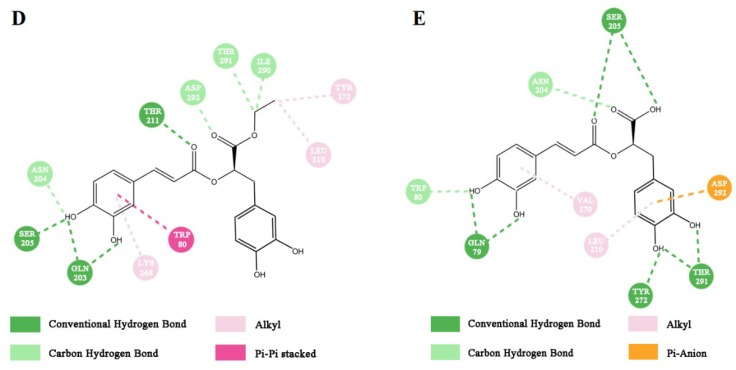
(**A**) Effect of RAE on Akt phosphorylation in endothelial cells treated with high glucose. EA.hy926 cells were treated with RAE or RA in the medium containing 33 mM of glucose for 48 h. Cells were exposed to LY294002 for 30 minutes before incubation with high glucose and RAE. Then, the cell lysates were collected. The phosphorylation of Akt was detected by Western blot using a phosphor-specific Akt antibody, normalized to total Akt. The results were expressed as mean ± SD (n = 5), and one representative image of five individual experiments is shown. ^##^ *P* < 0.01, vs. control; ^*^ *P* < 0.05, ^**^ *P* < 0.01, vs. high glucose; ^&^ *P* < 0.05, ^&&^ *P* < 0.01, vs. RAE (10 μM) group. (**B**) Docked pose of RAE in the active site of Akt. (**C**) Docked pose of RA in the active site of Akt. (**D**) Docking interactions of RAE with proper residues in the active site of Akt. (**E**) Docking interactions of RA with proper residues in the active site of Akt.

To substantiate whether the PI3K/Akt signal pathway was involved in the anti-apoptosis effect of RAE, we examined the level of phosphorylated Akt. As shown in Figure 3C and Figure 4A, high glucose treatment significantly inhibited Akt phosphorylation and led to severe apoptosis. When cells were treated with RAE or RA, as expected, Akt phosphorylation increased, and the apoptosis rate simultaneously decreased. The treatment of RAE was more effective than the treatment of RA at the same concentration. In Figure 4A, cells co-cultured with LY294002 and RAE had a significant decrease in the expression of p-Akt compared with the treatment of RAE, indicating that LY294002 suppressed the auxo-action of RAE on Akt phosphorylation, which is in accordance with the results presented in Section 2.3 (Figure 3C). These results demonstrated that RAE enhanced PI3K/Akt pathway activation under high-glucose conditions to protect endothelial cells.

Furthermore, a molecular docking study was performed to analyze the interactions of RA and RAE in the active site of Akt. Homo sapiens Akt (PDB code: 4EJN) was chosen for docking studies according to report before [24]. The docked poses and docking interactions of RAE and RA in the active site of Akt are depicted in Figure 4B–E. According to the docking results, the carboxyl moiety of RA presented conventional hydrogen bonding with Ser205, and the ethyl moiety of RAE was detected as binding to Thr291 and Ile290 residues through a carbon hydrogen bond and binding to Tyr292 and Leu210 through an alkyl–alkyl bond in the active site of Akt. Overall, these possible interactions formed the basis of the potent promoting activity of RAE to Akt.

The docking score and Ki value of the poses above were given in Table 1. A lower docking score and smaller Ki value suggest more favorable poses and more potential in binding affinity [25]. RAE showed a −8.50 kcal/mol docking score and a 0.59 μM Ki value, while RA showed a −8.03 kcal/mol docking score and a 1.30 μM Ki value. The values predicted that RAE probably binds more intensely with Akt than RA. These results are consistent with biological studies that indicate that RAE plays a better role in promoting Akt activation than RA. However, there is a limitation in the molecular docking study; that is, it’s difficult to consider the catabolism of RAE in endothelial cells.

### 2.5. Effect of RAE on the Expression of Bcl-2 and Bax

**Figure 5:**
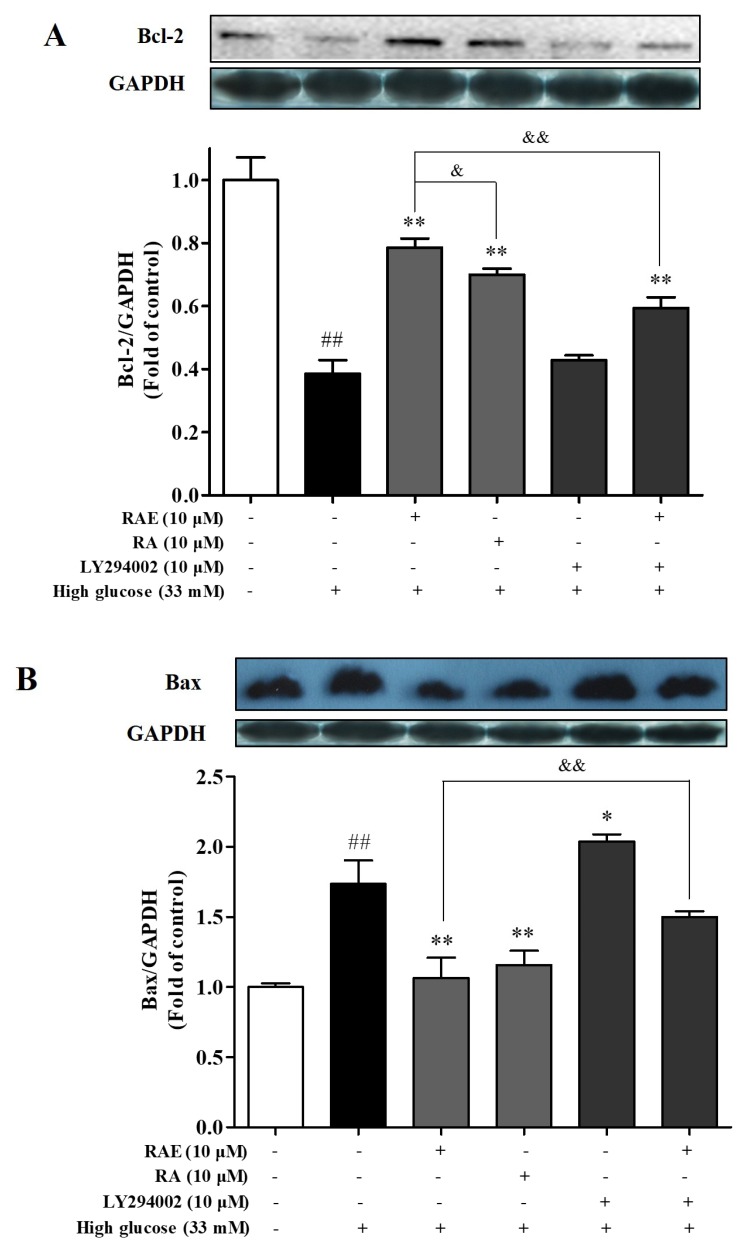
Effect of RAE on the expression of Bcl-2 and Bax in human endothelial cells treated with high glucose. EA.hy926 cells were treated with or without RAE (10 μM) or RA (10 μM) in the medium containing 33 mM of glucose for 48 h. Cells were exposed to LY294002 (10 μM) for 30 min before incubation with high glucose and RAE. The Bcl-2 (**A**) and Bax (**B**) expression were analyzed by Western blot with related antibodies. GAPDH was used as an internal control. The quantitative results were expressed as the mean ± SD (n = 3), and one representative image of three individual experiments is shown. ^#^ *P* < 0.05, ^##^ *P* < 0.01, vs. control; ^*^ *P* < 0.05, ^**^ *P* < 0.01, vs. high glucose; ^&^ *P* < 0.05, ^&&^ *P* < 0.01, vs. RAE (10 μM) group.

We also explored whether RAE regulated the apoptotic related proteins Bcl-2 and Bax via Western blot. Our data showed that high glucose suppressed the expression of the anti-apoptotic protein Bcl-2 (38.7% of control, Figure 5A), and increased the expression of the pro-apoptotic protein Bax (1.7-fold of control, Figure 5B). When cells were treated with RAE or RA, the balance between Bcl-2 and Bax was significantly recovered compared with the high-glucose model group. Interestingly, the treatment of RAE (10 μM) performed a greater promotion of Bcl-2 expression in human endothelial cells induced by high glucose than the treatment of RA (10 μM).

Moreover, when endothelial cells were co-cultured with RAE and LY294002 in high-glucose medium, the anti-apoptotic protein Bcl-2 was less expressed and the pro-apoptotic protein Bax was significantly increased compared with the treatment of RAE alone. It suggested that the expressions of Bcl-2 and Bax were affected by the PI3K/Akt pathway, and they might be downstream proteins of the PI3K/Akt pathway. These data supported that the anti-apoptotic effect of RAE was involved in the PI3K/Akt/Bcl-2 signal pathway.

### 2.6. Effect of RAE on the Expression of p-p65 and p-JNK

**Figure 6:**
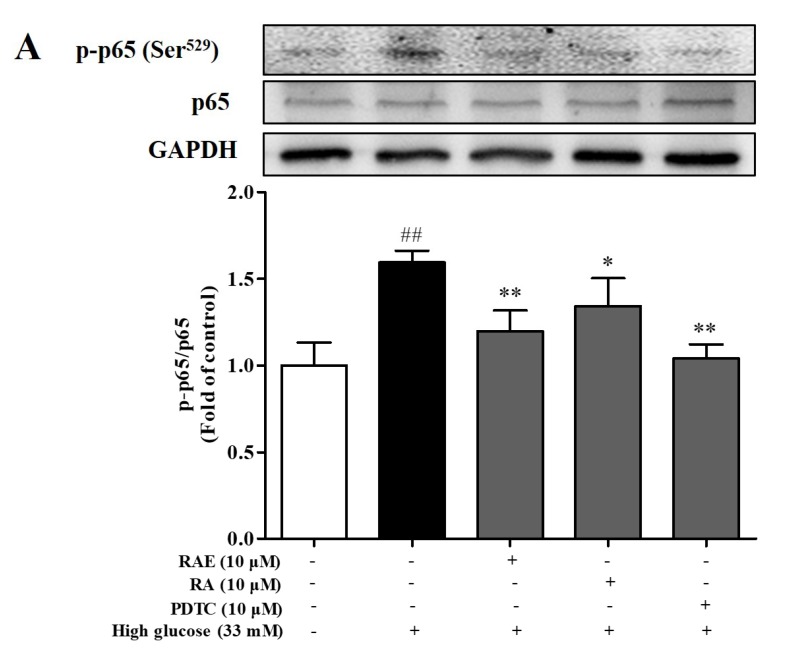

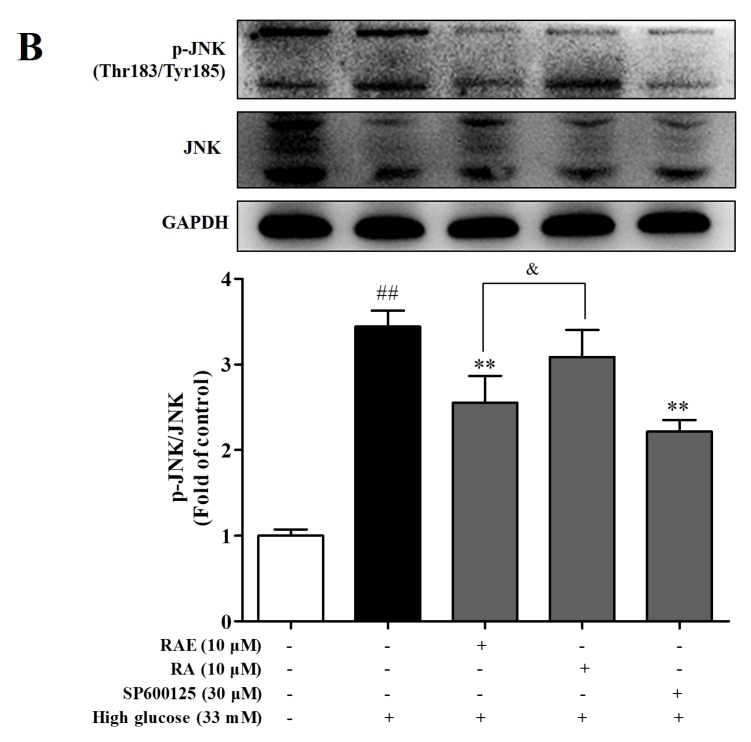
Effect of RAE on nuclear factor-κB (NF-κB) and c-Jun N-terminal kinase (JNK) activation in endothelial cells treated with high glucose. EA.hy926 cells were treated with or without RAE (10 μM) or RA (10 μM) in the medium containing 33 mM of glucose for 48 h. Cells were exposed to PDTC (10 μM) (**A**) and SP600125 (10 μM) (**B**) for 30 minutes before incubation with high glucose and RAE for 48 h. Then, the cell lysates were collected. The phosphorylation of NF-κB and JNK were detected by Western blot using a phosphor-specific related antibody. The results were expressed as mean ± SD (n = 5), and one representative image of five individual experiments was shown. ^##^ *P* < 0.01, vs. control; ^*^ *P* < 0.05, ^**^ *P* < 0.01, vs. high glucose; ^&^ *P* < 0.05, vs. RAE (10 μM) group.

We also examined the level of p-p65 and p-JNK to detect whether the NF-κB and JNK pathways were involved in the anti-apoptosis effect of RAE. As illustrated in Figure 3C, the apoptosis rate of treatment with PDTC (NF-κB inhibitor) (10 μM) significantly decreased, and the inhibition rate was 80.2%. Furthermore, as shown in Figure 6A, the level of p-p65 was significantly increased when incubated with high glucose for 48 h. Compared with the model group, the treatment of PDTC inhibited the phosphorylation of NF-κB-p65, and the inhibition rate was 86.7%. Treatment of RAE or RA also inhibited the activation of p65, and the inhibition rates were 71.1% and 48.9%, respectively.

Compared with the model group, the apoptosis rate of the endothelial cells decreased significantly (*P* < 0.05) when treated with SP600125 (JNK inhibitor) (10 μM), as shown in Figure 3C. The level of phosphorylated JNK in the SP600125 group was significantly decreased, and the inhibition rate was 51.4%, as shown in Figure 6B. The treatment of RAE also significantly inhibited the overactivation of JNK, and the inhibition rate was 45.3%. Additionally, RAE showed greater potency than RA in down-regulating JNK phosphorylation.

Combining Figure 3C with Figure 6, the data showed that the activation of NF-κB and JNK promoted cell apoptosis, indicating that the NF-κB pathway and JNK pathway were involved in high glucose-induced cell apoptosis and played positive roles. RAE suppressed the activation of p65/JNK to protect endothelial cells from injury when exposed to high glucose.

## 3. Discussion

Hyperglycemia is a common clinical metabolic disorder in diabetes that caused endothelial injury, which resulted in vascular complications [13]. In recent years, a large number of epidemiological and clinical studies have shown that the development of diabetic complications is directly related to increased glucose in body fluids [26,27]. In this study, we investigated the protective effect of RAE on vascular endothelial cells injury induced by high glucose and its mechanisms. EA.hy926 cells were chosen for the study, as they possess many of the characteristics of vascular endothelial cell functions such as angiogenesis, inflammation, and the containment of Weibel–Palade, which was found only in vascular endothelial cells [28]. We assessed cell viability by MTT assay for the initial evaluation of the protective effect of RAE on high glucose-induced injury in human endothelial cells. The results showed that both RAE and RA could significantly improve endothelial cell viability under a high-glucose environment in a dose-dependent manner.

High glucose leads to ROS production, which is one of the causes of vascular damage in diabetes mellitus. A causal link between elevated glucose and hyperglycemic damage is the increased production of superoxides by the mitochondrial electron transport chain, according to recent studies [29,30]. Intracellular ROS is one of the superoxides that is mainly produced by mitochondria and overproduces under high glucose conditions, which contributes to endothelial injury and ultimately causes apoptosis [31]. We evaluated intracellular ROS production by the fluorescence of 2′,7′-dichlorofluorescin diacetate (DCFH-DA) to detect the effect of RAE on oxidative stress. We found that RAE could markedly inhibit the overproduction of ROS, which is closely related to cell apoptosis and also presented dose dependence.

Mitochondrion is the major site of intracellular biological oxidation, and plays an essential role in apoptosis [32]. The mitochondrial apoptosis pathway is affected by the Bcl-2 family members, including anti-apoptotic protein Bcl-2, pro-apoptotic protein Bax, etc. Ultimately, the effector caspases are activated, leading to the mitochondrial membrane permeabilization and the release of cytochrome C, which then binds to Apaf-1. This complex triggers its oligomerization, forming an apoptosome and recruiting pro-caspase-9. The activated pro-caspase-9 subsequently activates caspase-3, which cleaved target proteins, further leading to the cell apoptosis [33]. As shown in our results, both RAE and RA could significantly down-regulate the expression of pro-apoptotic protein Bax, up-regulate the expression of anti-apoptosis protein Bcl-2, and inhibit the activity of caspase-3 in endothelial cells damaged by high glucose.

PI3K/Akt/Bcl-2 signaling is considered to be the anti-apoptosis pathway according to previous studies [10,34]. Both in vitro [5] and in vivo [35] experiments have proved that LY294002 blocks the drug effects of protecting endothelial cells from high glucose-induced damage. Activated Akt will directly phosphorylate BAD (a pro-apoptotic Bcl-2-family member), which inhibits the expression of Bcl-2 and other anti-apoptotic Bcl-2 family members; then, it protects cells from apoptosis [36]. In our study, cells incubated with LY294002 showed as high an apoptosis rate as the model group, and the effects were significantly reversed by RAE treatment. Furthermore, Western blot analysis showed that high glucose reduced the phosphorylation of Akt, while RAE could activate the phosphorylation of Akt under the high-glucose condition. This effect was reversed when exposed to LY294002, indicating that RAE reduced endothelial cells apoptosis through the PI3K/Akt pathway. Additionally, when cells were incubated with LY294002 in high glucose, the anti-apoptotic protein Bcl-2 was low expressed, and the pro-apoptotic protein Bax was significantly increased compared with the model group. The treatment of RAE significantly balanced the Bax/Bcl-2 ratio under the condition of inhibitor existence, which indicated that RAE activated the PI3K/AKt/Bcl-2 pathway.

The NF-κB pathway has a two-way effect of inhibiting apoptosis and promoting apoptosis, which possibly depends on cell types and stimulating factors [37,38]. However, the specific mechanism is not fully understood. The role of the NF-κB pathway in the promotion of apoptosis in endothelial cells induced by high glucose was recently investigated [9,39]. Here, we found that the NF-κB pathway was overactivated under high glucose conditions. The expression of p-p65 and the apoptosis rate were reduced when cells were exposed to PDTC, which indicated that the NF-κB pathway was involved in endothelial cells’ apoptosis caused by high glucose, and played a promoting role. When cells were incubated with RAE, the expression of p-p65 and the apoptosis rate decreased, confirmed that RAE inhibited endothelial cells apoptosis through inhibiting the NF-κB pathway.

JNK is a member of the mitogen-activated protein kinases (MAPKs) family [40]. MAPKs have been found to have four subtypes: extracellular regulated protein kinase (ERK1/2), p38MAPKs, JNK, and ERK5, which regulate a variety of physiological processes such as cell growth, differentiation, and apoptosis [41]. In recent years, studies have found that the JNK signaling pathway plays an important role in regulating apoptosis, but the effects remain controversial, which are related to cell types and stimulating factors [42]. Accumulating studies have demonstrated that the JNK pathway is activated in endothelial cells, and induces apoptosis under stimulation [43]. When JNK is activated, it is transferred to the nucleus and phosphorylates its major downstream substrate, c-jun. Then, it activates a transcription-dependent apoptotic signaling pathway [41]. In our study, high glucose induced an abnormal increase in the expression of p-JNK, and when treated with the SP600125, the p-JNK level was efficiently decreased. The results showed that JNK played a promoting role in the apoptosis of endothelial cells caused by high glucose. When cells were incubated with RAE, the expression of p-JNK and the apoptosis rate were decreased. These suggested that RAE protected endothelial cells from injury through the JNK pathway.

Importantly, these mechanisms may be interrelated. For example, hyperglycemia-induced oxidative stress promotes both NF-κB and JNK activation, and depresses PI3K activation [44,45,46]. NF-κB may also cross-talk with the JNK signaling pathway in high glucose-induced endothelial cell apoptosis [9]. In our present experiments, we did not verify the interactions among these pathways, which will be the next research task: to clarify the mechanisms and the potential target of RAE.

Our results indicated that RAE could significantly attenuate the damage of endothelial cells induced by high glucose, and showed greater potency than RA in terms of ROS generation, cell apoptosis, and proteins (including Bcl-2, p-Akt, and p-JNK) expression. As for its reason, firstly, we suppose that RAE enters the cell more easily than RA, since some ester drugs are beneficial for cell penetration. Secondly, the data obtained from molecular docking showed that the ethyl moiety of RAE was detected binding to the active site of Akt through carbon hydrogen and alkyl–alkyl bonds; these may be the basis for enhancing Akt activity.

**Figure 7:**
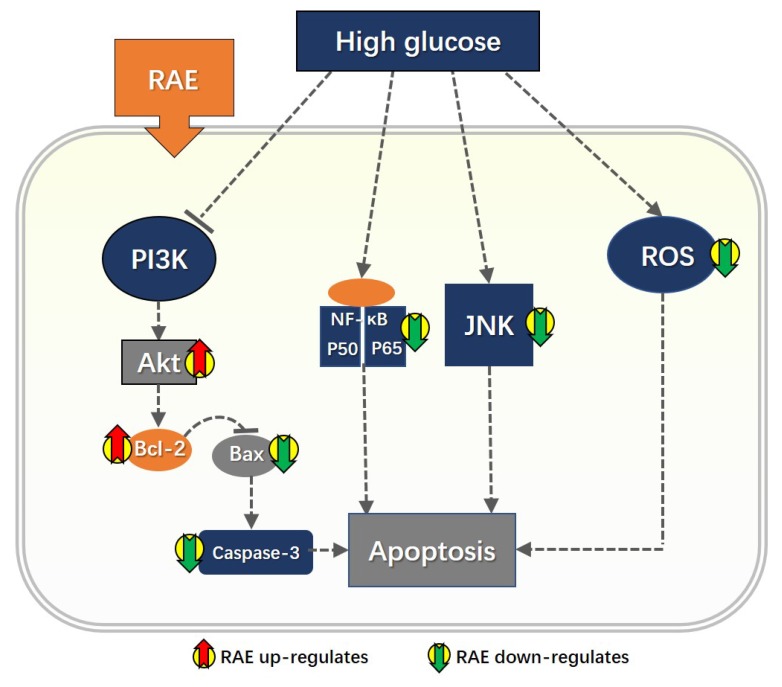
High glucose stimulated reactive oxygen species (ROS) production and promoted the activity of caspase-3. It also inhibited Akt activation, broke the balance between Bcl-2/Bax, and activates NF-κB and JNK activation, which are all accompanied by a higher apoptosis rate in endothelial cells compared with the normal group, indicating that these pathways were closely related to the apoptosis of endothelial cells under high-glucose conditions. Furthermore, the treatment of RAE could significantly reverse the effects caused by high glucose. Thus, RAE might have a close relationship with these pathways, which were involved with high glucose-induced apoptosis. However, the interactions among these pathways were not clarified.

In summary, our results proved that RAE could significantly attenuate the damage of endothelial cells induced by high glucose, and also indicated that the underlying mechanisms of RAE were partly involved with the PI3K/Akt/Bcl-2 pathway, the NF-κB pathway, and the JNK pathway, which suggested the potential target of RAE, as summarized in Figure 7. These results may promote further exploration to elucidate the mechanism and interaction between ingredients in anti-diabetes herbs, and eventually contribute to the development of treatments for diabetes complications.

## 4. Materials and Methods

### 4.1. Plant Materials

CC was collected from Putian city, Fujian province, China and identified by Assoc. Prof. Sheban Pu from the Department of Pharmacognosy at China Pharmaceutical University. RAE was extracted and isolated from CC according to methods reported before [16]. Rosmarinic acid was provided by Prof Jingyu Liang in the Department of Natural Medicinal Chemistry at China Pharmaceutical University.

### 4.2. Reagents

Low-glucose Dulbecco’s modified Eagle medium (L-DMEM) and fetal bovine serum (FBS) were purchased from Gibco (Grand Island, NY, USA). 3-(4,5-dimethylthiazol-2yl-)-2,5- diphenyltetrazoliumbromide (MTT), LY294002, PDTC, and SP600125 were obtained from Sigma-Aldrich (St. Louis, MO, USA). 2′,7′-dichlorofluorescin diacetate (DCFH-DA, a fluorescence probe for detection of ROS production), an enhanced chemiluminescence (ECL) kit, and a bicinchoninic acid (BCA) protein assay kit were purchased from the Beyotime Institute of Biotechnology (Shanghai, China). The Apoptosis fluorescence Hoechst 33258 assay kit, the Annexin V-FITC/PI apoptosis detection kit, and the Caspase-3 colorimetric assay kit were purchased from KeyGen Biotech Co., Ltd. (Nanjing, China).

A standard sample of protein molecular weight (Blue Plue II Protein Marker, 10–170 kDa) was purchased from Fermentas. Polyvinylidene difluoride (PVDF) membrane was purchased from Millipore (Billerica, MA, USA). Anti-Akt, anti-p-Akt, anti-Bcl-2, anti-Bax, anti-JNK, and anti-p-JNK were obtained from Cell Signaling Technology. Anti-NF-kB p65, anti-p-NF-kB p65, and GAPDH polyclonal antibody were purchased from Bioworld Technology (St. Paul, MN, USA). Anti-cleaved-Caspase-3 and HRP-labeled Goat IgG were purchased from Affinity Biosciences (Cincinnati, OH, USA). Other reagents are commercially available analytically pure products.

### 4.3. Cell Culture

Human endothelial cell line EA.hy926 were obtained from Professor Baolin Liu in the Department of Pharmacology for Chinese Materia Medica at China Pharmaceutical University. Human umbilical vein endothelial cell lines (HUVECs) were purchased from KenGEN Bio TECH Corp., Ltd. (Nanjing, China). Cells were cultured in L-DMEM containing 10% FBS, supplemented with 100 U/mL penicillin and 100 U/mL streptomycin at 37 °C with a gas mixture of 5% CO_2_ and 95% air. Culture medium was changed every day when cells were confluence. Endothelial cells in actively growing condition were seeded into plates for experiments.

### 4.4. Cell Viability Assay

High-glucose induced cell damage on EA.hy926 cells was used to evaluate the cytoprotecting effect of RAE. EA.hy926 cells (1 × 10^4^ cells/well) were seeded into 96-well plates. After 24 h, cells were treated with high glucose (33 mM D-glucose), while the control group was kept untreated. Then, these cells were co-cultured with RAE (1, 3, and 10 μΜ), RA (1, 3, and 10 μM) and Vit-C for 72 h, respectively. Subsequently, 20 µL of MTT solution (5 mg/mL) was added to each well and further incubated for four hours at 37 °C. Thereafter, the supernatant was removed, and 150 μL DMSO was added to dissolve the formazan crystals. The absorbance was measured at 490 nm by Thermo Scientific Varioskan Flash. The assay was repeated for three independent experiments and three replicates per experiment and the viabilities of treated groups were expressed as percentages of the control group, which was assumed to be 100%.

### 4.5. Detection of Intracellular ROS Production

Intracellular ROS production was monitored by flow cytometry using a DCFH-DA fluorescent probe. ROS in cells oxidize fluorescent DCFH to produce fluorescent DCF, which can be detected. Endothelial cells (1 × 10^5^ cells/well) were seeded into six-well plates. Cells were treated with high glucose (33 mM of D-glucose), while the control group was kept untreated. Then, the high-glucose exposed cells were co-cultured with RAE (1, 3, and 10 μΜ) or RA (10 μM), respectively. After incubation of 48 h, cells were washed twice by serum-free medium. Subsequently, cells were incubated with DCFH-DA (10 μM) for 20 min with serum-free medium at 37 °C, and harvested in PBS. Then, fluorescent intensity was measured by a FACSCalibur Flow Cytometer (BD Biosciences, San Jose, CA, USA) at an excitation wavelength of 488 nm and an emission wavelength of 535 nm.

### 4.6. Cell Morphology Assays by Hoechst Staining

Morphology of apoptotic cells was detected by nuclear staining with Hoechst 33258. Cells were treated with RAE (1, 3, and 10 μM), RA (10 μM), Vit-C (100 μM), RAE (10 μM) co-cultured with LY294002 (10 μM), LY294002 (10 μM), PDTC (10 μM), and SP600125 (30 μM) exposed to high glucose, respectively. LY294002, PDTC, and SP600125 were added into the medium 30 min before incubation with high glucose, respectively. After incubated for 48 h, cells were stained with Hoechst 33258 at a final concentration of 10 mg/L for 20 min at 37 °C. Stained cells were imaged at 340 nm using a fluorescent microscope (Olympus IX81, Tokyo, Japan).

### 4.7. Analysis of Apoptosis by Annexin V-FITC/PI Staining

In order to investigate the effect of RAE on cell apoptosis, Annexin V-FITC/PI staining was used. Cells were treated with RAE (1, 3, and 10 μM), RA (10 μM), Vit-C (100 μM), RAE (10 μM) co-cultured with LY294002 (10 μM), LY294002 (10 μM), PDTC (10 μM), or SP600125 (30 μM) exposed to high glucose, respectively. LY294002, PDTC, and SP600125 were added into the medium 30 min before incubation with high glucose, respectively. Endothelial cells (1 × 10^5^ cells/well) were seeded into six-well plates and incubated for 48 h. The apoptotic rate was analyzed by the Annexin V-FITC/PI apoptosis detection kit, according to manufacturer’s protocol. In brief, cells were digested and collected by trypsin without EDTA (Ethylenediaminetetraacetic acid) and washed by PBS twice. Then, cells were resuspended with 500 μL of binding buffer and mixed with 5 μL of Annexin V-FITC and 5 μL of PI (propidium iodide). After standing for 15 min at room temperature in the dark, the cells were detected by FACSCalibur Flow Cytometer immediately. Data analysis was performed using Flowjo7.6 software.

### 4.8. Caspase-3 Activity Assay

Caspase-3 activity was measured using a caspase-3 colorimetric assay kit based on the ability of caspase-3 to change acetyl-Asp-Glu-Val-Asp p-nitroanilide (Ac-DEVD-pNA) into a yellow formazan product (p-nitroanilide (pNA)) following the manufacturer’s instructions. Cells were treated with high glucose (33 mM of D-glucose), while the control group was kept untreated. Then, the high-glucose exposed cells were treated with RAE (1, 3, and 10 μΜ), RA (10 μM), or Vit-C (100 μM), respectively for 48 h; then, cells were lysed in a hypotonic buffer. The supernatants were collected and incubated with 200 μM of the substrate Ac-DEVD-rNA for two hours at 37 °C. Samples were measured at an absorbance of 405 nm.

### 4.9. Analysis of Western Blot

EA.hy926 cells or HUVECs were seeded in six-well plates until 80% confluency, and incubated with 33 mM of glucose in the presence of multifarious concentrations of tested sample or inhibitors for 48 h. Cells were washed with PBS once followed by homogenizing with 150 μL of ice-cold Lysis buffer per well, supplemented with one mM of phenylmethanesulfonyl fluoride. Protein was obtained by centrifugation at 15,000 g for 15 min at 4 °C, and the concentration of protein was measured with a BCA protein assay kit in accordance with the manufacturer’s protocol. Equal amounts of protein was separated by SDS-PAGE gel, which was prepared by 10% separation gel and 5% concentration gel, respectively, and transferred to PVDF membranes at 350 mA. The membranes were blocked for two hours at room temperature in 5% non-fat milk dissolved with TBST (Tris-buffered saline containing 0.05% Tween-20). Thereafter, the membranes were incubated with primary antibody (1:1000 dilution) at 4 °C overnight, and then washed and reacted with secondary antibody (1:2000 dilution) for two hours at room temperature. Finally, the bands of protein were developed by an enhanced chemiluminescence (ECL) detection system, and the resulting images were analyzed by Image J software (National Institutes of Health, USA).

### 4.10. Molecular Docking Analysis

Docking experiments were performed using an Auto-Dock Tool 4.2 [47], which was a suite of automated docking tools. In our present work, RAE and RA were docked to the active site of Akt kinase. The crystal structure of Akt kinase was retrieved from Protein Data Bank server (PDB code: 4EJN). First of all, the non-essential molecules such as water and ligand were removed from the Akt enzyme, and the energy of both the protein and ligand was minimized. The grid has been set at the center of the active site pocket and followed by an Autogrid run. Docking simulation has been repeated three times with similar parameters to improve the precision level of the results. The interactions were studied in terms of ligand interactions, binding score (Kcal/mol), and Ki value (μM).

### 4.11. Statistical Analysis

All of the data are expressed as mean ± SD from three or five independent experiments. Statistical differences were assessed by the one-way analysis of variance (ANOVA) and two-tailed Student’s t-test. Values of *P* < 0.05 were considered statistically significant.

## Figures and Tables

**Table 1:** Docking score and Ki value results of RAE and RA for Akt kinase.

Compound	Docking Score (kcal/mol)	Ki Value (μM)
RAE	−8.50	0.59
RA	−8.03	1.30
